# An Adult Case of Congenital Pulmonary Airway Malformation Undergoing Right Lower Lobectomy with Preoperative Arterial Embolization

**DOI:** 10.70352/scrj.cr.25-0796

**Published:** 2026-04-18

**Authors:** Kei Matsubara, Asuka Aizawa, Yutaka Hirano, Yuho Maki, Akihisa Tamura, Toshiya Fujiwara

**Affiliations:** 1 Department of Thoracic Surgery, Hiroshima City Hiroshima Citizens Hospital, Hiroshima, Hiroshima, Japan; 2 Department of Radiology, Hiroshima City Hiroshima Citizens Hospital, Hiroshima, Hiroshima, Japan

**Keywords:** congenital pulmonary airway malformation, arterial embolization, interventional radiology

## Abstract

**INTRODUCTION:**

Congenital pulmonary airway malformation (CPAM) is a rare developmental anomaly of the fetal tracheobronchial tree. Surgical resection is the primary treatment for CPAM. However, surgery for adult CPAM presents unique challenges due to the potential for severe pleural adhesions, increased risks of excessive blood loss, and prolonged surgical time. This report describes an adult case of CPAM managed with preoperative arterial embolization.

**CASE PRESENTATION:**

A 26-year-old male underwent a right lower lobectomy for CPAM with the aid of preoperative arterial embolization. Due to the prolonged course of CPAM, significant pleural adhesions and feeding arteries from the intercostal and phrenic arteries were anticipated. Preoperative embolization of the feeding arteries was performed to minimize the intraoperative bleeding. This approach allowed safe surgical resection with minimal blood loss and no postoperative complications.

**CONCLUSIONS:**

This case highlights the utility of preoperative arterial embolization in the management of complex CPAM in adults.

## Abbreviations

%FEV1
: % forced expiratory volume in 1 second
%VC
: % vital capacity
CCAM
: congenital cystic adenomatoid malformation
CPAM
: congenital pulmonary airway malformation
CTA
: CT angiography

## INTRODUCTION

CPAM, previously termed CCAM, is a rare developmental anomaly of the fetal tracheobronchial tree that is characterized by dysplastic cystic lesions originating from adenomatous hyperplasia of the bronchiolar epithelium.^1)^

The primary treatment for CPAM is surgical resection, to confirm the diagnosis and prevent complications, such as recurrent infections and malignant transformation.^2)^ However, surgery for adult CPAM cases presents unique challenges due to the potential for severe pleural adhesions, which often result from repeated infections over time.^3)^ This report presents an adult case of CPAM managed with preoperative arterial embolization to mitigate the intraoperative risks and achieve successful surgical outcomes.

## CASE PRESENTATION

**Fig. 1:**
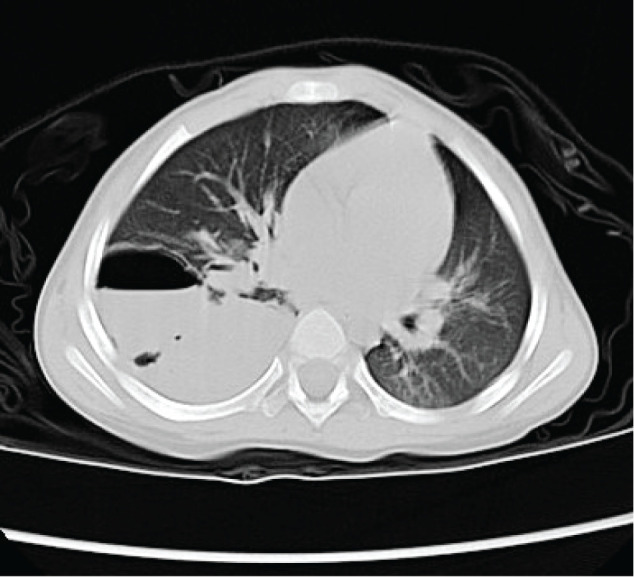
CT image of the right lower lobe abscess at the age of 1 year.

A 26-year-old male with a history of pediatric asthma and a lung abscess requiring chest drainage at the age of 1 year (**Fig. 1**) was referred to our department for thoracic surgery. At the age of 19 years, he was incidentally diagnosed with suspected CPAM based on routine chest radiography. However, his family preferred a follow-up examination to surgical treatment at that time. Although asymptomatic for 7 years, he developed an intermittent low-grade fever, persistent cough, and difficulty lying in the supine position. These symptoms prompted further evaluations.

**Fig. 2:**
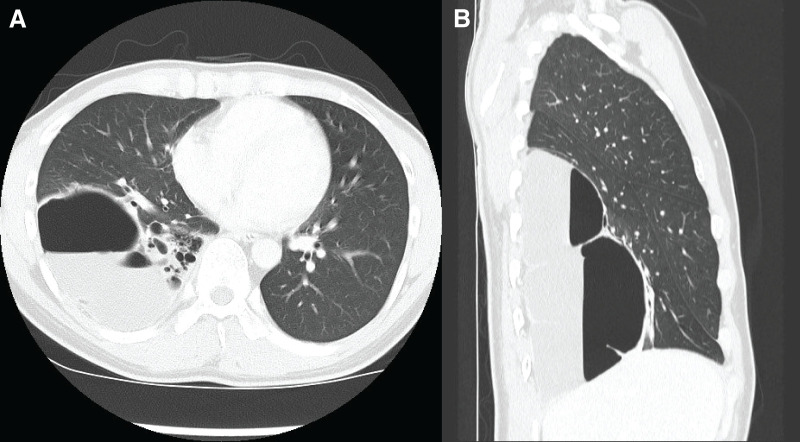
CT image of multiple cystic lesions in the right lower lobe at the age of 26 years. (**A**) Axial view and (**B**) sagittal view.

**Fig. 3:**
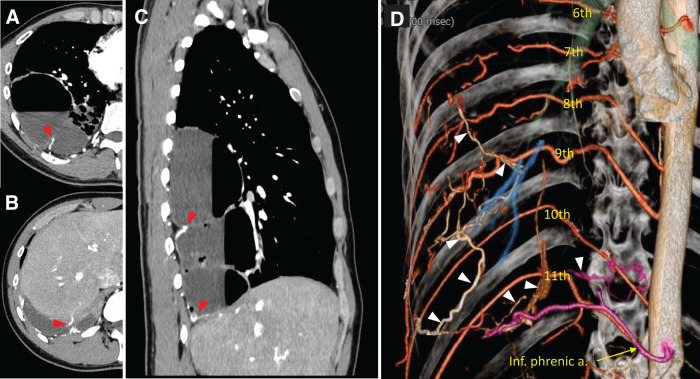
(**A**–**C**) Aberrant vascularization flowing into the lung parenchyma from the chest wall, with enhanced blood flow in the cyst walls. (**D**) 6–11th intercostal arteries and the Inf. phrenic a. Aberrant serpentine feeding arteries (arrow heads) originating from the intercostal arteries and the inferior phrenic artery are detected on CT angiography. Inf. phrenic a., inferior phrenic artery

CT revealed multiple cystic lesions in the right lower lobe; the largest lesion measured 9 cm in diameter and contained an air-fluid level (**Fig. 2**). Serpentine feeding arteries originating from the 6th to 11th intercostal arteries and the inferior phrenic artery were identified using CTA (**Fig. 3**). This aberrant vascularization flowed into the lung parenchyma from the chest wall, indicating severe adhesions. The flow was enhanced in the cyst walls. The sagittal view showed that the cysts occupied the majority of the right lower lobe, compressing the normal lung parenchyma. Pulmonary function tests revealed a %VC of 58.1% and a %FEV1 of 57.1%.

**Fig. 4:**
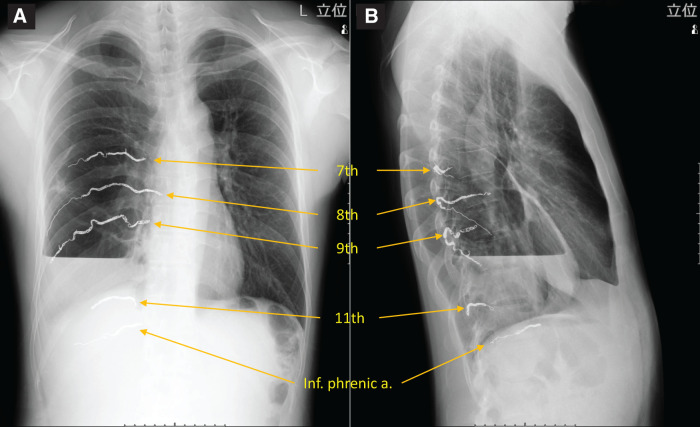
(**A**, **B**) Chest radiography after arterial embolization of the 6–11th intercostal and Inf. phrenic as. Embolic microcoils are visible, but gelatin sponge pledgets cannot be detected. Inf. phrenic a., inferior phrenic artery

Given the anticipated challenges of severe pleural adhesions and significant intraoperative bleeding, we planned a preoperative arterial embolization in collaboration with the radiology department. After obtaining informed consent, arterial embolization was performed 2 days prior to surgery. A 4-Fr catheter was inserted into the right femoral artery. Preliminary angiography of the arterial branches (6th to 11th intercostal arteries and the inferior phrenic artery) was performed individually. Aberrant vascularization was detected in all branches to varying degrees and involving smaller arteries that were not detected on CTA. Considering the degree of aberrant vascularization, bloodstream size, and risk of embolic materials flowing to the pulmonary vein, microcoils (Interlock; Boston Scientific, Marlborough, MA, USA; POD Packing Coil; Penumbra, Alameda, CA, USA) and/or gelatin sponge pledgets approximately 2 × 2 × 2 mm in size (Serescue; Astellas, Tokyo, Japan) were chosen to occlude the feeding arteries (**Fig. 4**).

**Fig. 5:**
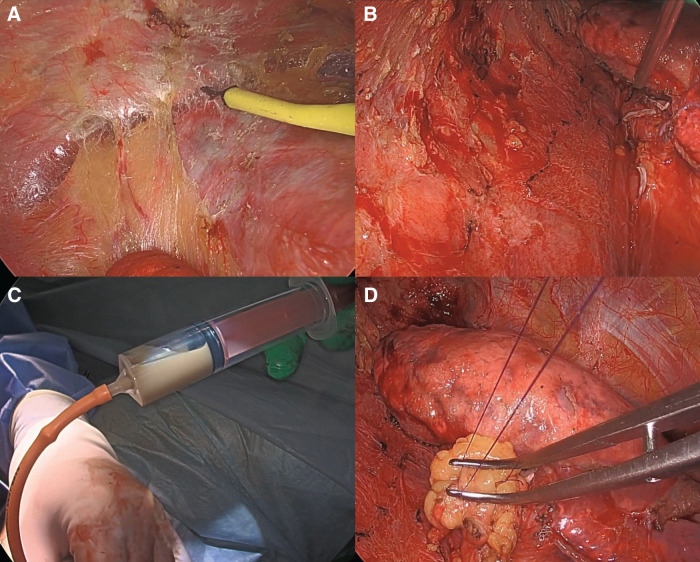
(**A**) Several fine feeding vessels are visible within the adhesions. (**B**) Bleeding from the chest wall is effectively controlled using energy devices. (**C**) Fluid aspiration from the cysts and (**D**) bronchial stumps are reinforced with pedunculated pericardial fat tissue.

**Fig. 6:**
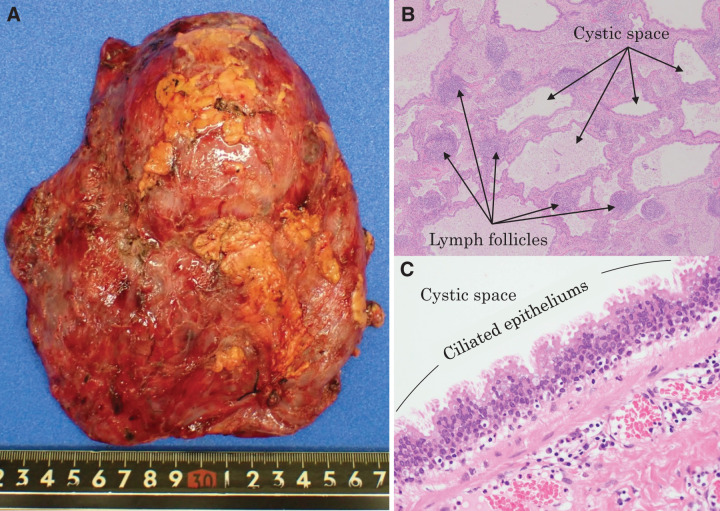
Pathological findings. (**A**) Macroscopic image and (**B**) microscopic examination shows some smooth muscle tissue and lymph follicles in the surrounding interstitial tissue. (**C**) High-power field image showing cyst cavities covered with partially serrated ciliated epitheliums.

Surgery was performed via an 8-cm posterolateral thoracotomy. Intraoperatively, the entire right lung was found to be densely adherent to the thoracic cavity. Adhesiolysis was carefully performed, and several fine feeding vessels were identified within the adhesions. Bleeding from the chest wall was effectively controlled using energy devices, and fluid aspiration from the cysts facilitated a clear surgical field. The bronchial stumps were reinforced with the pedunculated pericardial fat tissue (**Fig. 5**). The operative duration was 355 min, with a total blood loss of only 80 mL. The chest drain was removed on POD 4, and the patient was discharged on POD 7, without complications. Histopathologically, large cysts surrounded by groups of smaller cysts were observed (**Fig. 6**). The cyst cavities were covered with a partially serrated ciliated epitheliums. Smooth muscle tissue and lymph follicles were observed in the surrounding interstitial tissue, which is a characteristic of the bronchus. Based on the histopathological findings and clinical course, the diagnosis was compatible with CPAM, without evidence of malignancy. This case was considered type I CPAM. The postoperative course of the patient was uneventful for up to 6 months.

## DISCUSSION

CPAM accounts for approximately 25% of congenital lung anomalies, with an estimated incidence of 1 in 25000–35000 births.^4)^ While the condition is commonly detected during the prenatal or neonatal period, some cases remain undiagnosed until adulthood and are often discovered incidentally or during evaluation for complications such as infection or pneumothorax.^5,6)^ Adult patients typically present with symptoms such as fever, cough, dyspnea, or recurrent pulmonary infections. In our case, the patient developed intermittent fever and cough after years of being asymptomatic.

Stocker’s classification system defines 5 CPAM types based on the histopathological features.^1)^ Type I, the most common subtype that accounts for 60%–70% of all cases, comprises large cysts up to 10 cm lined by pseudostratified ciliated cells interspersed with mucous cells.^2)^ Although rare, malignant transformation is predominantly associated with type I and IV lesions.^6)^ Consequently, surgical resection is recommended for symptomatic CPAM or asymptomatic cases with a high malignancy risk.

Adult cases often pose unique surgical challenges due to severe pleural adhesions and the development of feeding vessels from adjacent structures, owing to the prolonged disease course. Zeng et al. reported that 71.7% of adult patients with CPAM exhibited pleural adhesions, possibly leading to longer operative times and increased intraoperative blood loss.^3)^ In our case, preoperative CT imaging revealed feeding arteries from the intercostal and inferior phrenic arteries, necessitating a strategy to mitigate potential bleeding.

Preoperative arterial embolization has been utilized to reduce the intraoperative blood loss in various pulmonary conditions, such as aspergilloma, chronic expanding hematoma, and tuberculosis.^7–9)^ In a study of pulmonary aspergilloma cases, embolization significantly reduced the mean blood loss (676.47 vs. 1264.58 mL).^7)^ Similarly, Kuwata et al. demonstrated that arterial embolization improved the safety of surgical resection in chronic expanding hematoma cases.^8)^ In a study of patients who underwent surgery for tuberculosis-destroyed lungs, the preoperative arterial embolization group demonstrated that shorter operative times corresponded to lower amounts of intraoperative bleeding and rates of postoperative complications, including pulmonary infection, anemia, and hypoproteinemia, compared to the direct surgical treatment group.^9)^ These findings support the efficacy of embolization in managing challenging pulmonary surgeries. In our case, the operative time was not shortened. However, arterial embolization successfully reduced the intraoperative bleeding, enabling a safer surgical resection. Aberrant vascularization was detected even on CT. In particular, the 9th and 11th intercostal arteries were more dilated than the bilateral arteries. These findings indicated a large bloodstream with aberrant vascularization. Although several feeding vessels remained within the adhesions, bleeding was effectively controlled using energy devices. Preoperative arterial embolization is recommended when multiple aberrant vascularizations flowing into the lung parenchyma from the chest wall are observed.

A considerable complication of arterial embolization is vessel injury and bleeding from the insertion site. In particular, cerebral infarction caused by perfusion of embolic materials through the pulmonary vein and spinal cord ischemia caused by embolization of the artery of Adamkiewicz should be considered in the pulmonary field. When a dangerous anastomosis is detected on the preliminary angiograms, embolization should only be performed distally to the origin of the spinal feeder.^10)^ To our knowledge, this is a rare report of an adult CPAM case managed with preoperative arterial embolization for severe adhesions and aberrant vascularization. This approach was effective in minimizing bleeding and overcoming the surgical challenges posed by severe adhesions and aberrant vascularization.

## CONCLUSIONS

This case highlights the utility of preoperative arterial embolization as a surgical adjunct in the management of adult CPAM. Arterial embolization can be a valuable strategy in complex cases involving severe adhesions and feeding vessels by reducing intraoperative bleeding and facilitating safer surgical resection. Multidisciplinary collaboration and careful preoperative planning are essential for achieving optimal outcomes in such challenging cases.

## DECLARATIONS

### Availability of data and materials

The datasets supporting the conclusions of this article are included within the article.
